# Supplementary figures and images for: Structure of human phagocyte NADPH oxidase in the resting state
Source: eLife. 2022 Nov 22;11:e83743. doi: 10.7554/eLife.83743 (PMC9711523; doi:10.7554/eLife.83743)

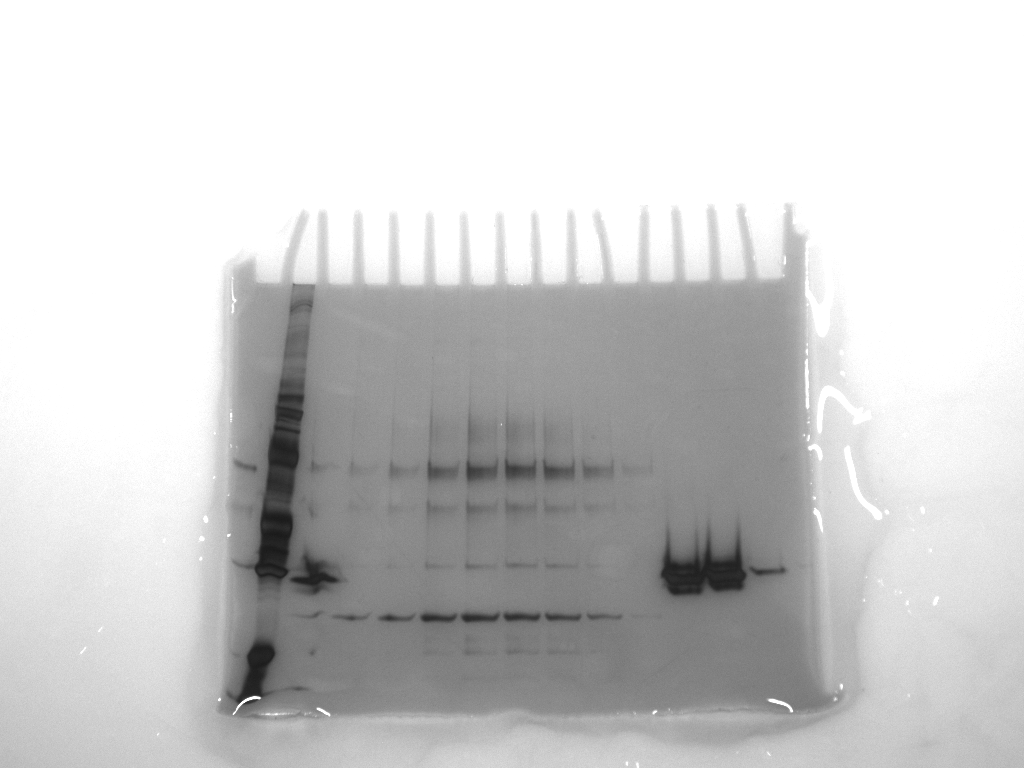

Supplement: Figure 1—figure supplement 1—source data 1. [file elife-83743-fig1-figsupp1-data1.zip › Figure 1-figure supplement 1-source data 1/full raw unedited gels.docx]

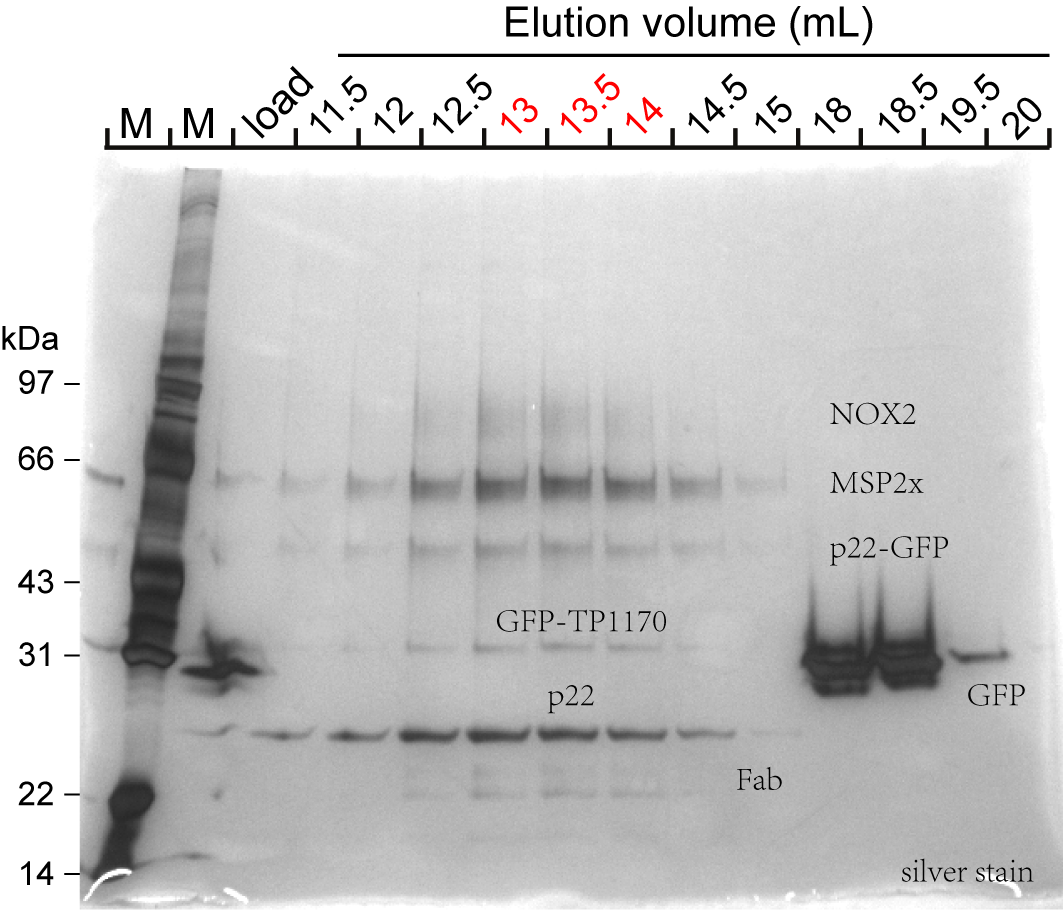

Supplement: Figure 1—figure supplement 1—source data 1. [file elife-83743-fig1-figsupp1-data1.zip › Figure 1-figure supplement 1-source data 1/uncropped gels.docx]
